# Preparation and Characterization of Porous Poly(Lactic Acid)/Poly(Butylene Adipate-Co-Terephthalate) (PLA/PBAT) Scaffold with Polydopamine-Assisted Biomineralization for Bone Regeneration

**DOI:** 10.3390/ma15217756

**Published:** 2022-11-03

**Authors:** Kullapop Suttiat, Wassanai Wattanutchariya, Chawan Manaspon

**Affiliations:** 1 Biomedical Engineering Institute, Chiang Mai University, Chiang Mai 50200, Thailand; 2 Department of Prosthodontics, Faculty of Dentistry, Chiang Mai University, Chiang Mai 50200, Thailand; 3 Advanced Manufacturing and Management Technology Research Center, Department of Industrial Engineering, Faculty of Engineering, Chiang Mai University, Chiang Mai 50200, Thailand

**Keywords:** polymeric bone scaffold, mussel-inspired polydopamine, biomimetic mineralization, bone regeneration, osteogenesis

## Abstract

The development of scaffolds that simultaneously provide porous architectures and osteogenic properties is the major challenge in tissue engineering. Herein, a scaffold with high porosity and well interconnected networks, namely poly(lactic acid)/poly(butylene adipate-co-terephthalate) (PLA/PBAT), was fabricated using the gas foaming/ammonium bicarbonate particulate leaching technique. Mussel-inspired polydopamine (PDA)-assisted biomineralization generated by two-step simple soaking in dopamine solution and 10× SBF-like solution was performed to improve the material’s osteogenicity. Highly porous scaffolds available in less organized opened cell structures with diameters ranging from 10 µm to 100 µm and 200 µm to 500 µm were successfully prepared. The well interconnected porous architectures were observed through the whole thickness of the scaffold. The even deposition of the organic–inorganic bioactive mineralized layer composed of PDA and nano-scale hydroxyapatite (HA) crystals on the scaffold surface was evidenced by scanning electron microscopy (SEM), energy-dispersive X-ray analysis (EDX), Fourier transform infrared spectroscopy (FTIR), and X-ray diffraction (XRD). The developed scaffold exhibited high total porosity (84.17 ± 1.29%), a lower surface contact angle (θ = 45.7 ± 5.9°), lower material degradation rate (7.63 ± 2.56%), and a high level of material biocompatibility. The MTT assay and Alizarin Red S staining (ARS) confirmed its osteogenic enhancement property toward human osteoblast-like cells (MG-63). These results clarified that the developed porous PLA/PBAT scaffold with PDA-assisted biomineralization exhibited good potential for application as a biomaterial for bone tissue regeneration and hard tissue engineering.

## 1. Introduction

An alteration of the alveolar ridge contour following natural tooth loss compromises the esthetics and function of dental treatments, especially at the anterior part of the jaw [1]. The management of the tooth extraction site via the tissue regenerative approach is of interest as a new strategy in dental treatment. Tooth socket filling with conventional bone grafts to preserve the alveolar bone contour has been accepted as a reliable and effective approach to control and minimize the alveolar ridge change following natural tooth loss [2].

The conventional bone substitute materials, including the autograft, allograft, and xenograft, have been considered the materials of choice for decades [3]. However, the invasive and complicated surgical operations required for autograft bone harvesting, as well as the risk of immune rejection or disease transmission through the utilization of the allogenic or xenogenic bone graft, are significant drawbacks for clinical application [4,5]. The recent advances in biotechnology have enabled the development of various types of biomaterials for medical and dental regenerative approaches. Bioceramics and natural and synthetic biopolymers, as well as their composites, provide a strong possibility to be utilized as alternative materials to conventional bone grafts in medical and dental scenarios [6]. Among them, biodegradable polymers and their composites are considered promising candidates for tissue engineering due to their biocompatibility and biodegradative behaviors. They also possess high versatility in manufacturing and design. In addition, their properties can be easily modified to meet specific requirements through the adjustment of their chemical compositions or material structures [7].

The tissue scaffold should function as a template for cells during the tissue regenerative process. It must have permeability to facilitate cell growth, migration, and nutrient flow. It is generally accepted that adequate porosity with appropriate pore sizes, as well as interconnectivity between each porous structure in the scaffold, are essential for bone regeneration. Scaffolds with inadequate porosity result in the restriction of cell migration and nutrient distribution, including waste product removal. On the other hand, a scaffold with a large pore structures leads to a reduction in the specific surface area, which plays a major role in cell adhesion, proliferation, and extracellular matrix formation [8]. 

Nowadays, extensive studies on highly porous scaffolds made from natural or synthetic biopolymers, including their composites, as a bone cell template for tissue engineering are attracting increasing interest. A wide range of natural polymers, such as collagen, chitosan, and synthetic polymers including polylactic acid (PLA), polyglycolic acid (PGA), polycaprolactone (PCL), and polyethylene glycol (PEG), have been extensively researched [9]. Compared to natural polymers, the physicochemical and biological properties of synthetic polymers are more controllable, reproducible, and predictable. In addition, their properties can be conveniently modified through the engineering of polymer chain segments or polymeric functional groups [10]. 

Among the most promising candidates for synthetic biopolymers, PLA is the most attractive polymer for bone tissue engineering. It shows basic properties that are suitable for utilization in different biomedical applications, such as good mechanical characteristics, high thermal resistance, appropriate biodegradation, as well as excellent biocompatibility [11,12]. However, the material’s brittleness, the lack of cell recognition sites, and the absence of osteoconductivity are significant drawbacks that restrict the material’s application in tissue regeneration [13].

According to a previous article, the incorporation of a high-toughness compatible biopolymer such as polybutylene adipate-co-terephthalate (PBAT) into the PLA matrix can effectively improve the material’s toughness and flexibility [14]. This high-toughness polymer has been commonly used for packaging or agricultural purposes [15,16]. Only a few studies have mentioned it as a biomaterial for tissue regenerative applications. Recent studies addressed the in vitro biocompatibility of a PLA/PBAT blend toward fibroblast cells. However, they concluded that the lack of bioactive properties was a significant drawback for its application clinically [17,18]. To overcome this limitation, surface coating with bioactive substances in the inert polymer matrix has been considered as one of the simplest processes to improve the material’s bioactivity.

Hydroxyapatite (HA) is a calcium phosphate (CaP)-based material that is commonly applied as a bioactive substance to promote the bone cells’ compatibility and enhance the osteogenic potential of bio-inert materials [19]. The inductive effect on new bone formation and its similarity in chemical composition to the inorganic components of natural bone make HA suitable for use as a bioactive component in bone biomaterials [20]. Besides the enhancement effect toward bone cells due to the existence of the HA component in the polymer matrix, the surface micromorphology of the biomaterial has also been accepted as another crucial factor that plays a significant role in the material’s bioactivity. The high surface-to-volume ratio of the scaffold provides a significant improvement in the material’s hydrophilicity and cell viability. The increase in the surface roughness as well as the alternation of the surface morphological features due to the deposition of HA nanocrystals also ensured the responsiveness and viability of bone cells [21,22]. For these reasons, the modification of the porous scaffold surface with nanoscale hydroxyapatite crystals could favor both cell bioactivity and the material’s osteogenic property at the same time. Furthermore, the alternation of the surface morphology of the PLA/PBAT porous structure due to the deposition of a nanoscale CaP biomineralized layer could also provide an enhancement effect on cell adhesion, proliferation, and differentiation.

Various techniques have been proposed for the development of nanoscale HA crystals on the surfaces of porous biomaterials. Biomimetic mineralization by two steps of simple dipping in simulated body fluid (SBF) solution is one of the most simple and effective techniques to deposit nano HA crystals on the entire surface of the porous specimen without interfering with the bulk material properties [23]. Regarding this technique, an organic layer that is suitable for the nucleation, growth, and orderly formation of inorganic crystals is initially fabricated. Then, inorganic deposition is performed and results in the formation of a bioactive organic–inorganic hybrid layer on the outer surface of the substrate [23,24]. 

**Figure 1:**
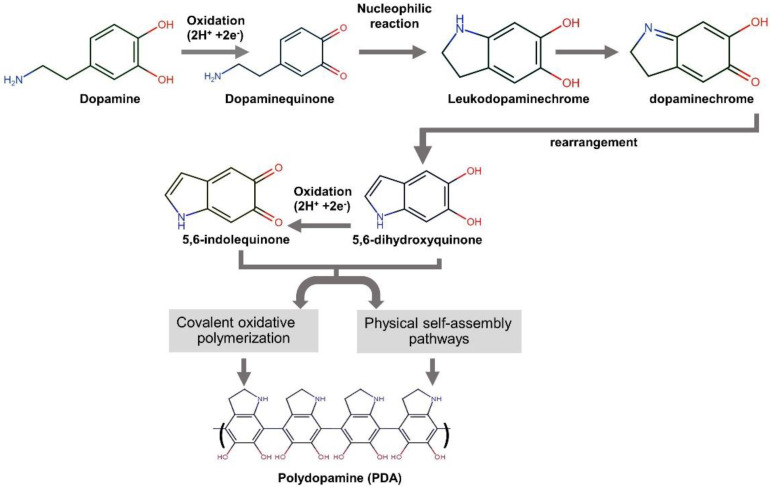
A schematic illustration of the reaction mechanism of polydopamine formation from dopamine [21,26,27].

The mussel-inspired polydopamine (PDA), which contains catechol, amine, and imine functional groups, has been recommended as an organic platform to immobilize bioactive molecules or ions onto various types of surfaces [25]. This strong and reliable PDA thin film on the surface substrate is generated through base-triggered oxidation and dopamine self-polymerization during the material’s soaking in dopamine solution [26]. Figure 1 exhibits the mechanism of PDA formation from the aqueous dopamine solution. It is commonly accepted that the intermediate indoles, 5,6-hydroxyindole and 5,6-indolequinone, play a significant role in the formation of the dopamine-based polydopamine. The PDA structure has heterogeneity. It comprises oligomers of covalently bonded dimers and higher oligomers of 5,6-hydroxyindole and 5,6-indolequinone held by charge transfer and hydrogen bonding [27].

**Figure 2:**
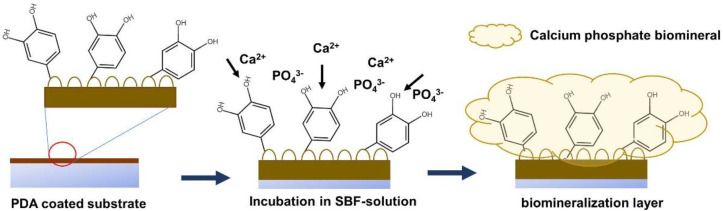
A schematic illustrating the procedure of biomineral deposition on a PDA-coated surface.

The formation of the CaP biomineralized layer on the PDA-modified surface is initiated by the deposition of calcium (Ca^2+^) and phosphate (PO_4_^3−^) ions on the PDA-coated surface through the accelerating effect of the surface negative charge induced by the catechol and amine functional groups on the PDA layer. The progression of the layer-by-layer deposition of Ca^2+^ and PO_4_^3−^ ions on the PDA-modified surface results in the formation of the CaP biomineralized layer, covering the surface of the substrate [22,23,24]. A schematic of the biomineralization process is provided in Figure 2.

Simulated body fluid (SBF) at various concentrations can be used as a source of Ca^2+^ and PO_4_^3−^ ions for surface modification using the biomimetic biomineralization technique. The non-aggressive conditions in terms of pH and the deposition temperature allow this technique to be performed on materials with heat or acid/base sensitivity. In addition, this surface modification technique is also successfully performed on materials with high porosity or complicated structures, because of the excellent penetration ability of the SBF solution [28,29,30].

In this study, a porous PLA/PBAT scaffold with well-interconnected networks and an osteogenic property was prepared by the gas foaming/ammonium bicarbonate particulate leaching method; then, the material’s bioactive property toward human osteoblast-like cells (MG-63) was improved by PDA-assisted biomineralization. The general morphology, the surface properties, and the in vitro osteogenic property were characterized. We hypothesized that the developed porous PLA/PBAT scaffold with PDA-assisted biomineralization could provide an enhancement effects toward the bone regenerative process, and that it could also exhibit potential for application as an alternative biomaterial in bone tissue engineering.

## 2. Materials and Methods

### 2.1. Materials

The PLA (PLA 4032D) and the PBAT (Ecoflex F blend C1200) were supplied by NatureWorks (Plymouth, MN, USA) and BASF SE (Ludwigshafen am Rhein, Germany), respectively. Ammonium bicarbonate, dopamine hydrochloride, Tris (hydroxymethyl) aminomethane (Tris), and phosphate-buffered saline (PBS) were supplied by Sigma Aldrich (St. Louis, MO, USA). Sodium chloride (NaCl), potassium chloride (KCl), calcium chloride dihydrate (CaCl_2_·2H_2_O), magnesium chloride hexahydrate (MgCl_2_·6H_2_O), sodium dihydrogen phosphate (NaH_2_PO_4_), sodium bicarbonate (NaHCO_3_), and dichloromethane were obtained from Union Science (Chiang Mai, Thailand). All reagents were analytical-grade and used as received.

### 2.2. Fabrication of Porous PLA/PBAT Scaffold

The porous PLA/PBAT scaffold was prepared by the gas foaming/particulate leaching technique [31]. Briefly, a 20% (*w*/*v*) PLA/PBAT solution was prepared by dissolving 20 g of PLA and PBAT pellets at the ratio of 70:30 in 100 mL of dichloromethane (DMC) under agitation at 80 rpm for 6 h. Then, 15 g of sieved ammonium bicarbonate (NH_4_HCO_3_) particles of an average diameter of 200–250 µm as the effervescent agent was mixed with 7 mL of PLA/PBAT blend solution. The resulting homogeneous gel paste was cast into a petri dish and left in the fume hood for 2 h to partially evaporate the solvent. Then, the semi-solidified mixture was immersed in 50 °C distilled water for 5 min to induce the decomposition of the ammonium bicarbonate particles into carbon dioxide (CO_2_) and ammonium gas (NH_3_). The resulting porous scaffolds were collected and rinsed with distilled water three times. The as-prepared scaffolds were soaked in an excessive amount of distilled water for 48 h and dried at 60 °C in a hot air oven for 24 h to eliminate the remaining solvent. 

### 2.3. Biomimetic Mineralization 

The spontaneous deposition of a PDA film on the surface of pristine porous PLA/PBAT scaffolds was achieved by immersing the scaffold in a dopamine solution (2 mg of dopamine hydrochloride per milliliter of 10 mM Tris HCl, pH 8.5) contained in an uncovered beaker at 37 °C for 24 h. To avoid the formation of PDA nanoparticles in the solution, agitation at 70 rpm was performed during the coating process. Amber staining on the porous PLA/PBAT scaffold was observed when the PDA formation occurred. The PDA-coated scaffolds were rinsed three times with deionized water to remove the unattached PDA molecules and air-dried at 45 °C for 4 h.

A stock solution of simulated body fluid with a 10-times concentration of calcium and phosphate ions (10× SBF-like solution) was prepared. The chemical reagents used to prepare 1000 mL of 10× SBF-like solution are given in Table 1. The chemicals were dissolved in 800 mL of distilled water in the order given, one by one, except NaHCO_3_. Following the addition of NaH_2_PO_4_, the solution was completed to 1000 mL with deionized water and then kept in a refrigerator at 4 °C until use.

**Figure 3:**
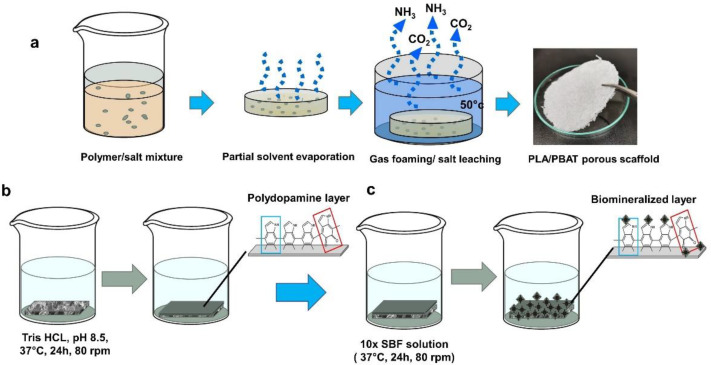
A schematic illustration of the preparation process of the porous PLA/PBAT scaffold with PDA-assisted biomineralization. (**a**) The fabrication of porous PLA/PBAT specimen via gas foaming/particulate leaching technique. (**b**) The formation of bioactive PDA layer on the surface of porous PLA/PBAT scaffold by soaking in dopamine solution. (**c**) The preparation of the biomimetic mineralized layer on PDA-coated film by soaking in 10× SBF-like solution.

For biomineralized deposition, 100 mL of 10× SBF-like stock solution was placed into a glass beaker; then, 0.084 g NaHCO_3_ was added to increase the hydrogen carbonate ion concentration to 10 mM. The PDA-coated scaffolds were soaked in 10× SBF-like solution and incubated at 37 °C under constant shaking at 70 rpm for 24 h. The resulting scaffolds with the biomimetic PDA-assisted biomineralization layer were collected and rinsed with deionized water to remove the unreacted ions and undesired salt molecules. The scaffolds were dried at 45 °C for 4 h and kept in a desiccator until use. The process of specimen preparation and surface modification is illustrated in Figure 3.

### 2.4. Specimen Characterization

#### 2.4.1. Scaffold Morphology

The scaffolds were sputtered with gold at a current of 50 mA for 60 s; then, the scaffold morphologies were investigated by scanning electron microscopy (SEM; JSM-IT300; JEOL, Tokyo, Japan) at an accelerating voltage of 15 kV. The surface chemical composition was identified by energy-dispersive X-ray spectroscopy (EDX).

#### 2.4.2. Physicochemical Characterization 

Small pieces of the PLA/PBAT, PLA/PBAT with PDA, and PLA/PBAT with PDA-assisted biomineralization scaffolds were pressed into plates and observed via attenuated total reflection–Fourier transform infrared spectra (ATR–FT-IR). The analysis was performed by Fourier transform infrared spectroscopy (FTIR, Nicolet 6700, Thermo Fisher Scientific, Waltham, MA, USA) at a spectrum ranging from 400 to 4000 cm^−1^, a resolution of 4 cm^−1^, and a number of scans of 64 times/min. 

The phase structures presented on the PDA-assisted biomineralized layer were identified by X-ray diffractometry (XRD) (SmartLab, Rigaku Co., Tokyo, Japan). Before XRD testing, the pristine PLA/PBAT, the PLA/PBAT scaffold coated with PDA, and the PLA/PBAT with PDA-assisted biomineralization were crushed and ground into fine powders. The XRD analysis was performed at 40 kV and a scanning of 4° min^−1^ ranging from 10° to 60° using Cu K α radiation. 

#### 2.4.3. Mechanical Properties

The porous PLA/PBAT scaffolds with and without PDA-assisted biomineralization were prepared in cylindrical shapes of 10 mm diameter and 5 mm thickness with plane parallel ends. The length and diameter were measured using a digital caliper. The compressive test was performed according to the process described in the previous article [33] that was based on the ASTM D695-96 standard. The compressive strength was evaluated by a universal testing machine (Instron 5566, Instron Co., Norwood, MA, USA) at a crosshead speed of 0.5 mm/min using a 10 kN load cell. The stress that was used to compress the scaffolds to 30% of their original height was determined as the compressive stress for porous scaffold specimens. The Young’s modulus (E) was obtained by fitting a tangent line to the initial part of the stress–strain plot. The gradient of this line is defined as the modulus of elasticity. Five samples were evaluated for each group, and the average values were recorded.

#### 2.4.4. Surface Contact Angle Measurements

Static contact angle measurement via the sessile drop method with a tensiometer (Attention Theta Flex, Biolin Scientific, Västra Frölunda, Sweden) was used to determine the material surface wettability. Here, 3 µL of deionized distilled water was deposited on the scaffold surface; then, the contact angle was immediately measured. 

#### 2.4.5. Porosity

The total porosity of scaffolds was determined by the gravimetric method, as described in the previous literature [34,35]. Five specimens from each group were weighed in air and results recorded as m_0_. Triplicate measurement was performed and the average value was recorded. The volume of each specimen was calculated. The porosity of open pores was calculated using Equation (1).

Porosity (%) = 1 − [(m_0_)/(V)/ρ _polymer_] × 100 (1)

where m_0_ was the initial dried mass of the scaffold, and V was the volume of each scaffold. The density of PLA was 1.24 g cm^−3^ [36] and the density of PBAT was 1.23 g cm^−3^ [37]. Then, the density of PLA/PBAT at the ratio 70:30 wt% referred to the density of PLA (1.24 g cm^−^^3^).

#### 2.4.6. Water Uptake

The in vitro study for determining the water uptake efficiency of the scaffolds was performed in phosphate-buffered saline (PBS) solution (pH = 7.4). Five specimens of 10 × 10 × 2 mm from each group were weighed and results recorded as W_0_. The measurement was performed in triplicate and the average value was recorded. Each scaffold was immersed in 10 mL PBS at 37 °C for 1, 7, 14, and 30 days. At each predetermined time point, the saturated scaffolds were removed from the solution and carefully wiped with wet filter paper to remove the excess water on the surface; they were then weighed and values recorded as W_1_. The percentage of water uptake was calculated by Equation (2) [17].

Water uptake (%) = [(W_1_ − W_0_)/W_0_] × 100 (2)

where W_1_ was the mass of the saturated material (g) and W_0_ was the initial weight of the dried specimen (g).

#### 2.4.7. In Vitro Degradation Test

A phosphate-buffered saline (PBS) solution without calcium and magnesium (pH = 7.33) was used as the soaking medium for the in vitro degradation test. The porous scaffolds (10 × 10 × 2 mm) were precisely weighed before and after soaking in 10 mL of the PBS solution at 37 °C under continuous 70 rpm agitation for predetermined periods. The measurement was performed in triplicate and the average value was used as the representative datum. At each predetermined time, the specimens (*n* = 5) were collected and washed with distilled water, and then subjected to lyophilization for 24 h. The dried scaffolds were weighed, and the percentage of weight loss was calculated by Equation (3) [38].

Weight loss (%) = [(W_1_ − W_0_)/W_0_] × 100 (3)

where W_0_ and W_1_ were the initial weight and weight at the degradation time point, respectively.

### 2.5. Biological Evaluation

The MG-63 human osteosarcoma cell line, purchased from the JCRB cell bank (Ibaraki City, Osaka, Japan), was used in this study. Dulbecco’s Modified Eagle Medium (DMEM) supplemented with 10% Fetal Bovine Serum (FBS) and 1% Antibiotic–Antimycotic (AA) represented the complete DMEM used for cell culture. The condition at 37 °C in a humidified atmosphere of 5% CO_2_ was determined as the standard condition for MG-63 cell incubation.

#### 2.5.1. Cell Viability and Biocompatibility

The methylthiazol tetrazolium (MTT) assay was employed according to the ISO 10993/EN 30993 standard for the qualitative analysis of cell viability and biocompatibility. Briefly, the scaffolds were sterilized by UV irradiation for 15 min on each side. The extracted media of the testing material were prepared at the specimen–medium ratio of 0.1 g/mL by incubating the sterilized scaffold in complete medium and then incubating it at 37 °C for 24 h. The scaffold was removed from the culture medium using a 0.22 µm cellulose acetate syringe filter.

The MG-63 cells were seeded on a 24-well plate at a density of 2 × 10^4^ cells/well (*n* = 3) and incubated at standard conditions for 24 h. Then, the complete medium was discarded and replaced with the material extraction medium. Following incubation for 24 h, 3 days, and 5 days, the metabolic activity of MG-63 cells was assessed by MTT assay. The MTT product, formazan, was measured by a microplate reader (Biosan Hipo MMP-96, Microplate Photometer, Biosan, Latvia) at 568 nm. The percentage of cell viability was calculated. The metabolic activity of cells cultured in complete DMEM and 1% TritonX-100 was used as the positive and negative control, respectively. Triplicate measurements were performed, and the average value was recorded as the representative datum.

#### 2.5.2. Cell–Scaffold Attachment

The UV-sterilized pristine PLA/PBAT and PLA/PBAT with PDA-assisted biomineralization scaffolds were impregnated with complete DMEM for 24 h, and then placed into a 24-well tissue culture plate. A suspension of MG-63 osteoblast-like cells (5 × 10^4^ cells) was directly seeded onto each scaffold. A sterilized glass-covered slide was used as a control group. The specimens were incubated under the standard conditions for 3 days. At the predetermined time, cells were fixed with 3% glutaraldehyde in PBS for 15 min and then dehydrated with a graded series of 30%, 50%, 70%, 90% and absolute ethanol solution for 5 min each. The dehydrated specimens were treated with hexamethyldisilazane (HMDS) for 15 min, dried at room temperature for 24 h, and sputtered with gold for SEM investigation.

#### 2.5.3. Osteogenic Differentiative Property

The osteogenic potential of the developed PLA/PBAT scaffold with PDA-assisted biomineralization was indirectly evaluated via the level of mineralization on MG-63 cells. Alizarin Red S staining was performed to quantify the amount of calcium formation in MG-63 cells after incubating with the specimen-extracted culture medium for 7, 14, and 21 days. The complete culture medium and the osteogenic culture medium (10 nM dexamethasone, 0.2 mM ascorbic acid, and 10 mM β-glycerophosphate in the completed culture medium) were designated as the negative and positive control, respectively.

At each predetermined time, the cultured medium was discarded. The cells were rinsed with PBS and then fixed with cold methanol (4 °C) for 15 min. The fixed cells were rinsed with distilled water and stained with 1% (*w*/*v*) Alizarin Red S for 3 min at 25 °C, followed by washing with deionized water 3 times to remove the excess staining agent. The stained cells were left to dry at room temperature for 5 h, and then investigated under a light microscope. To quantify the amount of Alizarin Red S staining, 400 µL of 10% (*w*/*v*) cetylpyridinium chloride monohydrate solution was added into each well to dissolve the stain. The absorbance of supernatant from each well was measured at 570 nm using a spectrophotometer (Tecan, Infinite 200 Pro, Männedrof, Switzerland).

### 2.6. Statistical Analysis

The IBM SPSS Statistics 24 (IBM corporation, New York, NY, USA) software was applied for independent *t*-test and one-way ANOVA (α = 0.05). The data were expressed as the mean ± SD. 

## 3. Results

### 3.1. Scaffold Morphology

**Figure 4:**
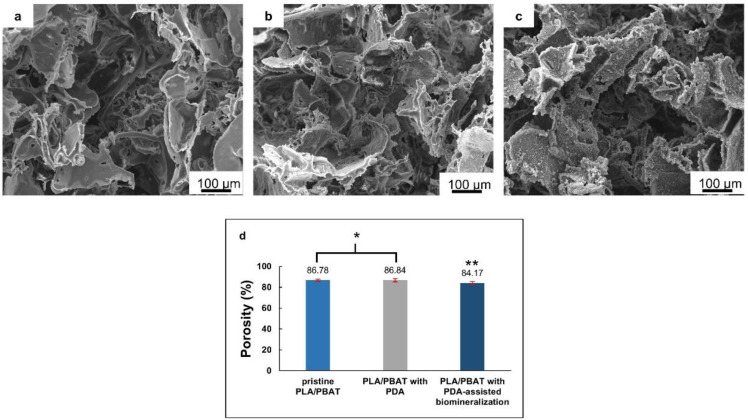
The scanning electron microscope (SEM) images of the pristine PLA/PBAT scaffolds (**a**), the PLA/PBAT scaffold with PDA surface modification (**b**), and the PLA/PBAT scaffold with PDA-assisted biomineralization (**c**). The percentage of porosity of the scaffold measured by the gravimetric method (**d**). The difference in number of asterisks represents statistical significance (*p* ≤ 0.05).

The overall morphological features of the pristine PLA/PBAT, PLA/PBAT coated with PDA, and PLA/PBAT with PDA-assisted biomineralization are presented in Figure 4. The SEM images of the pristine PLA/PBAT scaffold (Figure 4a) revealed the formation of three-dimensional open porous structures with interconnected networks on the surface and throughout the whole thickness of the scaffold. Less organized porous structures were observed. The irregularly shaped, open porous cells with different diameters were assessed. Microporous cells with a diameter ranging from 10 µm to 100 µm were present between the macropores, whose diameter ranged from 200 µm to 500 µm. The pristine PLA/PBAT scaffold did not show the formation of a dense skin layer on the outer surface, as usually observed on specimens fabricated via the conventional casting technique.

The morphological features of the scaffold with the bioactive PDA coating are presented in Figure 4b. The SEM images at low magnification did not show clear evidence of the deposition of the thin PDA film generated by the oxidization and self-polymerization of dopamine (DA) in air under mildly alkaline conditions (pH 8.5) on the surfaces of porous scaffolds. The morphological characteristics of the porous cells of the PLA/PBAT scaffold with PDA deposition were not obviously different compared to the pristine PLA/PBAT scaffold.

After surface modification by soaking in 10× SBF-like solution for 24 h, the formation of an irregular Ca/P mineralized layer on the surface of the porous scaffold was clearly exhibited (Figure 4c). The porous architectures and the interconnected networks within the scaffold could still be maintained following the CaP biomineral deposition.

The porosity (%) of the scaffolds, measured by the gravimetric method, is exhibited in Figure 4d. A non-significant difference in porosity (*p* ≥ 0.05) between the pristine PLA/PBAT scaffold and the PLA/PBAT scaffold with PDA coating was observed. The deposition of the CaP biomineralized layer on the outer surface of the scaffold resulted in a small but significant decrease in the scaffold’s porosity. However, the scaffolds in all groups exhibited porosity higher than 80%.

**Figure 5:**
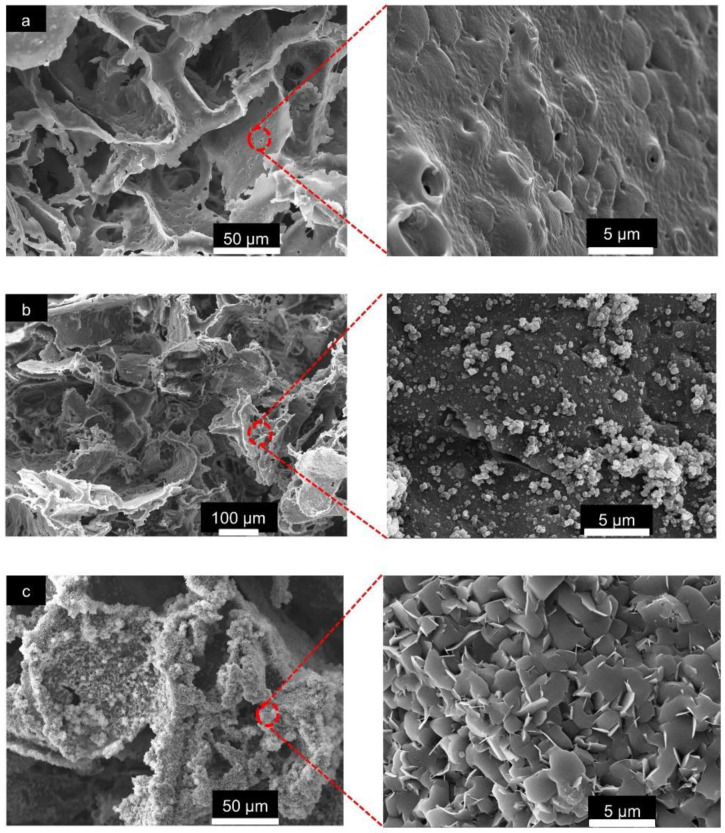
The SEM images of the surface of the pristine scaffold and the PLA/PBAT scaffold with PDA-assisted biomineralization at 500× and 5000×. The presentation of the micropores on the walls of the porous cell structures in pristine PLA/PBAT scaffold (**a**). The formation of a uniform PDA layer and some covalently bonded PDA particles and aggregates on the surface of the PDA-coated PLA/PBAT scaffold (**b**). The plate-like crystalline structure of the CaP biomineralized layer on the surface of the PLA/PBAT scaffold with PDA-assisted biomineralization (**c**).

The SEM images of the pristine PLA/PBAT scaffold at high magnification (5000×) revealed an irregular surface with approximately 1 to 2 µm diameter pores on the walls of the porous structure (Figure 5a). The even formation of the PDA film and aggregated PDA particles were observed in the PLA/PBAT scaffold with the PDA surface coating (Figure 5b). Following the surface modification by PDA-assisted biomineralization, the formation of nanoscale crystalline structures on the surface of the PLA/PBAT scaffold was observed. The SEM revealed the agglomeration of nanoscale plate-like apatite to form the CaP biomineral on the PLA/PBAT scaffold with the PDA bioactive layer (Figure 5c). The deposition of the CaP mineralized layer resulted in the disappearance of the micropores on the porous cell walls of the PLA/PBAT scaffold with PDA-assisted biomineralization.

**Figure 6:**
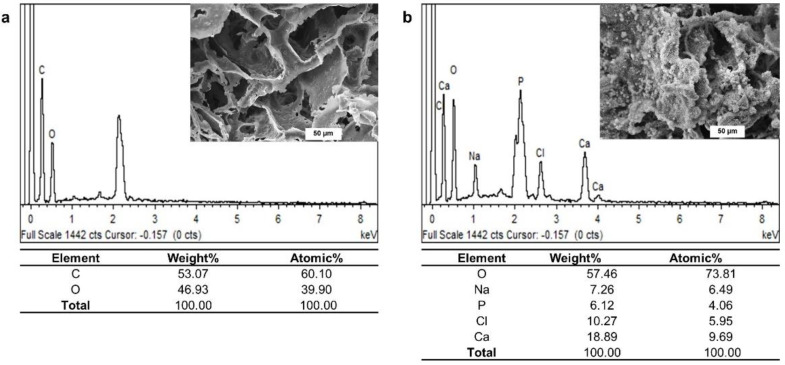
The energy-dispersive X-ray (EDS) spectra of the pristine PLA/PBAT scaffold (**a**) and the PLA/PBAT scaffold with PDA-assisted biomineralization (**b**).

The EDX spectrum confirmed the existence of oxygen (O) and carbon (C) as the main components of the pristine PLA/PBAT scaffold (Figure 6a). The additional presence of calcium (Ca), sodium (Na), chlorine (Cl), and phosphorous (P) on the EDX spectrum of PLA/PBAT with PDA-assisted biomineralization revealed the successful formation of the CaP biomineralized layer on the outer surface of the PLA/PBAT scaffold (Figure 6b). The deposited biomineralized layer provided the Ca/P atomic ratio of 2.38.

### 3.2. Physicochemical Characterization of the Scaffolds

#### Compressive Strength

**Figure 7:**
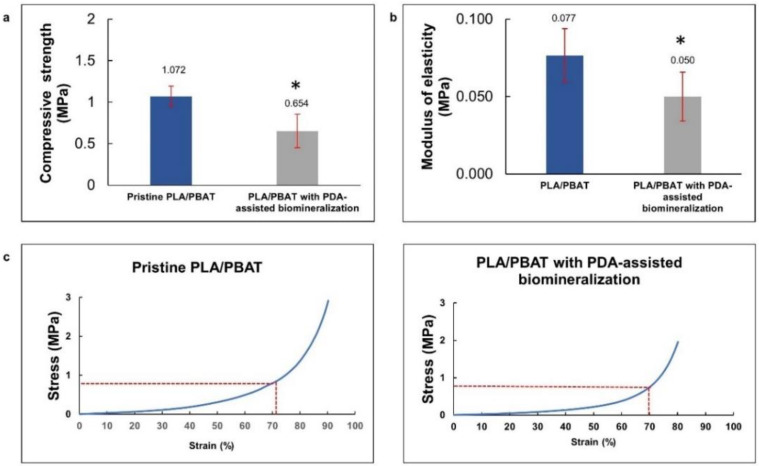
The average compressive stress (**a**) and average modulus of elasticity (**b**) of PLA/PBAT scaffold and PLA/PBAT scaffold with PDA-assisted biomineralization. The difference in number of asterisks represents statistical significance (*p* ≤ 0.05). The representative stress–strain curves for pristine PLA/PBAT scaffold and PLA/PBAT scaffold with PDA-assisted biomineralization are exhibited (**c**).

The compressive strength and modulus of elasticity of the pristine PLA/PBAT and PLA/PBAT scaffolds with PDA-assisted biomineralization surface modification, including the representative stress–strain curves, are exhibited in Figure 7a. The rapid increase in the stress–strain curves at the end of the test, observed in both groups, represented the compressive deformation without fracture or breakage of the porous architectures inside the scaffold. For this reason, the compressive strength of the scaffold was determined from the average stress that caused 70% scaffold deformation, as described in the previous literature [33]. The compressive strength and compressive modulus are presented in Figure 7b,c. A significantly lower compressive strength and modulus of elasticity were observed on the scaffold with PDA-assisted biomineralization. 

**Figure 8:**
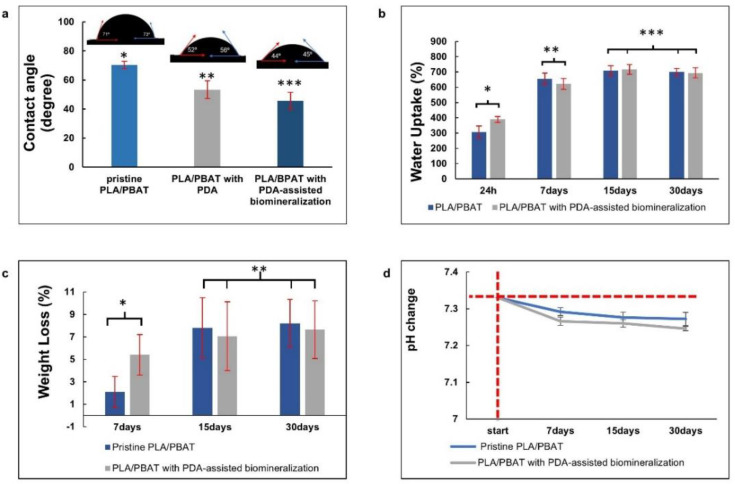
The in vitro degradation tests. The average water contact angles (**a**), the water uptake (%) (**b**), and the material weight loss (%) versus degradation time (**c**) of the PLA/PBAT scaffold with and without surface modification, and the pH alteration of the degradation medium (**d**). The vertical and horizontal red dashed lines represent the starting point of the investigating period and the initial pH of the soaking medium, respectively. The different numbers of asterisks indicate statistical significance (*p* ≤ 0.05).

The surface contact angles of the pristine PLA/PBAT, the PLA/PBAT with PDA coating, and the PLA/PBAT with PDA-assisted biomineralization are presented in Figure 8a. The scaffold with PDA-assisted biomineralization exhibited the lowest surface contact angle (45.7 ± 5.9), followed by the scaffold with PDA coating (53.5 ± 6.07) and the pristine PLA/PBAT scaffold, which yielded the highest value (70.4 ± 2.5). 

Figure 8b exhibits the water uptake (%) of scaffolds after soaking in PBS solution for 24 h, 7 days, 15 days, and 30 days. At 24 h and 7 days after soaking in PBS, an increase in water uptake with the increased observation time was observed. Then, adsorption equilibrium was achieved after day 15 of testing. The pristine PLA/PBAT scaffold and the PLA/PBAT scaffold with PDA-assisted biomineralization showed the ability to absorb water in the range of 306% to 840% and 389% to 848%, respectively. At each investigation period, a significant difference in the percentage of water uptake between the pristine scaffold and scaffold with surface modification was not observed (*p* ≥ 0.005). 

Figure 8c presents the material weight loss (%) after the in vitro degradation test in PBS at 37 °C for 7, 15, and 30 days. The material weight loss in both groups was increased with time at the initial stage, and then a linear relationship was observed. At each investigation time, the percentage of material weight loss of the scaffold with and without surface modification was not significantly different (*p* ≥ 0.05). After 30 days of the in vitro degradation test, the material weight loss of 8.19 ± 2.13% and 7.63 ± 2.56% was observed on the pristine PLA/PBAT scaffold and the PLA/PBAT scaffold with PDA-assisted biomineralization, respectively. All specimens showed morphological and structural integrity throughout the experiment. 

The pH alterations of the soaking medium (PBS) after the in vitro degradation test at each investigation time, which was associated with the release of degradation products from the amorphous phase of the PLA/PBAT blend, are exhibited in Figure 8d. A continuous reduction in the pH from 7.33 to around 7.2 was observed in both groups. A statistically significant result was not found regarding the pH value of the soaking medium of the PLA/PBAT scaffold with PDA-assisted biomineralization compared to the pH value of the soaking medium of the pristine PLA/PBAT scaffold.

### 3.3. Chemical Composition Analysis

#### 3.3.1. FTIR Analysis

**Figure 9:**
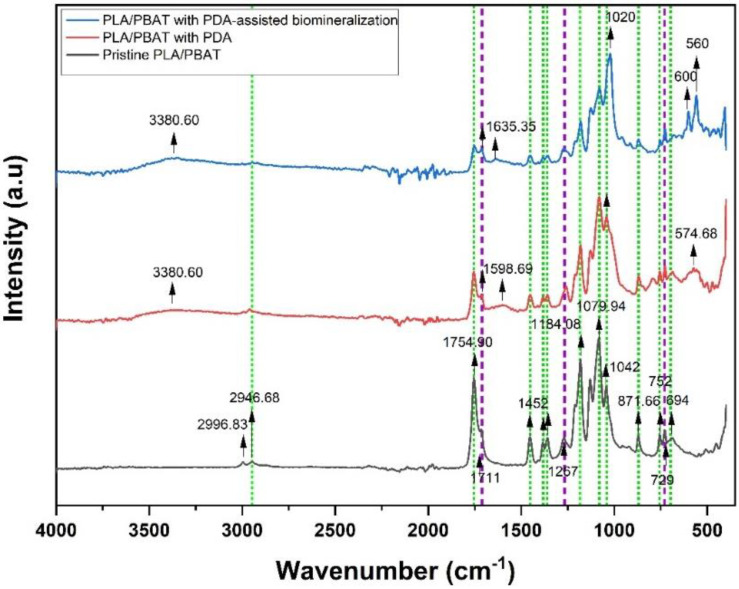
Fourier-transform IR (FTIR) spectra of pristine PLA/PBAT, PLA/PBAT with PDA, and PLA/PBAT with PDA-assisted biomineralization. The green and purple dashed lines indicate the characteristic bands of PLA and PBAT, respectively.

The ATR–FTIR spectrum taken in the reflection mode acquired from the pristine PLA/PBAT scaffold, the PLA/PBAT scaffold with PDA, and the PLA/PBAT with PDA-assisted biomineralization is displayed in Figure 9. Selected IR bands were labeled and assigned based on the reference infrared band tables [39] and previous works [40]. 

Considering the FTIR spectra of the PLA/PBAT blend, the bands of PLA and PBAT were taken into account. The PLA bands included (i) the asymmetric and symmetric stretching vibrations of the CH_3_ group present in the saturated hydrocarbons (2996.93 cm^−1^ and 2946.68 cm^−1^, respectively), (ii) the stretching vibrations of the C=O group (1754.90 cm^−1^), (iii) the asymmetric bending vibrations of the CH_3_ group (1452 cm^−1^), (iv) the deformation and symmetric bending vibrations of the CH group (1382 and 1360 cm^−1^, respectively), (v) the stretching vibrations of the C–O–C group (1184.08, 1079.94, and 1042 cm^−1^), and (vi) the band at 871 cm^−1^ and 752 cm^−1^ assigned to the amorphous phase and the crystalline phase of PLA, respectively.

In the case of PBAT, the characteristic peaks were (i) the stretching vibrations of the C=O group of the ester bond (1711 cm^−1^), (ii) the symmetric stretching vibrations of the C–O group (1267 cm^−1^), and (iii) the bending vibration absorption of the CH plane of the benzene ring (727 cm ^−1^). The appearance of characteristic bands of both PLA and PBAT in the spectrum of the pristine PLA/PBAT, the PLA/PBAT scaffold with PDA, and the PLA/PBT with PDA-assisted biomineralization indicated that the PLA/PBAT blend was not completely miscible.

After surface coating with PDA, three new absorbance signals were observed at 3380 cm^−1^, 1598.94 cm^−1^, and 574.68 cm^−1^. The broad peak at 3388 cm^−1^ was assigned to -OH bending, and the peak at 1598.94 cm^−1^ was attributed to the overlapped peaks of the C-C vibrations of the aromatic ring and the N-H bending vibrations. In addition, a broad peak at 574.68 cm^−1^ was a fingerprint area for dopamine that belonged to catechol stretching vibration [41]. Therefore, the FTIR spectra confirmed the presence of amine and phenolic hydroxyl groups that belonged to PDA on the surface via the coating process. These results indicated the successful coating of PDA on surface of the porous PLA/PBAT scaffold. Moreover, the reduction in the intensity of the PLA and PBAT characteristic peaks after PDA coating also confirmed the deposition of the PDA layer over the entire surface of the porous scaffold.

Focusing on the FTIR spectra of PLA/PBAT with PDA-assisted biomineralization, new absorbance peaks at 560, 600, and 1020 cm^−1^ that corresponded to the PO_4_^3−^ anionic group content [42,43] were observed, besides the characteristic peaks of the PLA/PBAT blend and PLA/PBAT with PDA coating. These findings confirmed the mixing of the PDA polymer and CaP minerals in the CaP biomineralized layer generated by soaking in 10×SBF-like solution. The intensity of the characteristic peaks of the PLA/PBAT blend was significantly decreased following the PDA-assisted biomineralization. This alteration could be explained by the coverage of the CaP biomineralized layer on the PLA/PBAT blend surface, which resulted in a masking effect on the expression of the characteristic peaks of the substrate underneath the surface coating layer.

#### 3.3.2. XRD Analysis

**Figure 10:**
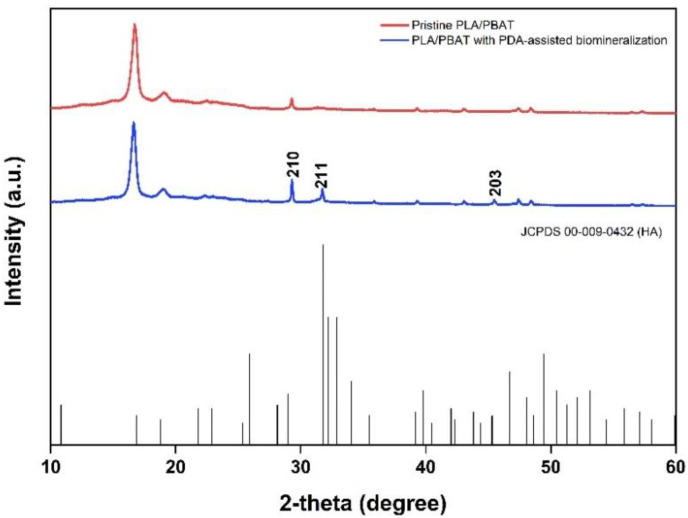
The XRD patterns of the pristine PLA/PBAT, the PLA/PBAT with PDA-assisted biomineralization layer, and the characteristic peaks related to hydroxyapatite based on JCPDS 00-009-0432.

The crystallinity of the biomineralized PLA/PBAT scaffold was investigated by XRD. Sharp diffraction peaks at the 2-theta angle of 31.774 and 48.624 were found in the XRD pattern of the PLA/PBAT scaffold with PDA-assisted biomineralization, as exhibited in Figure 10. Compared to the XRD pattern of the pristine PLA/PBAT, the scaffold with PDA-assisted biomineralization showed an increase in peak intensity at 29.3 degrees following the biomineralization surface modification. These three peaks at the 2-theta angles of 31.774, 48.624, and 29.3 degrees corresponded to the 210, 221, and 203 peaks of hydroxy apatite (JCPDS 00-009-0432).

### 3.4. Biological Evaluations

#### 3.4.1. Cell Viability and Cell Attachment

**Figure 11:**
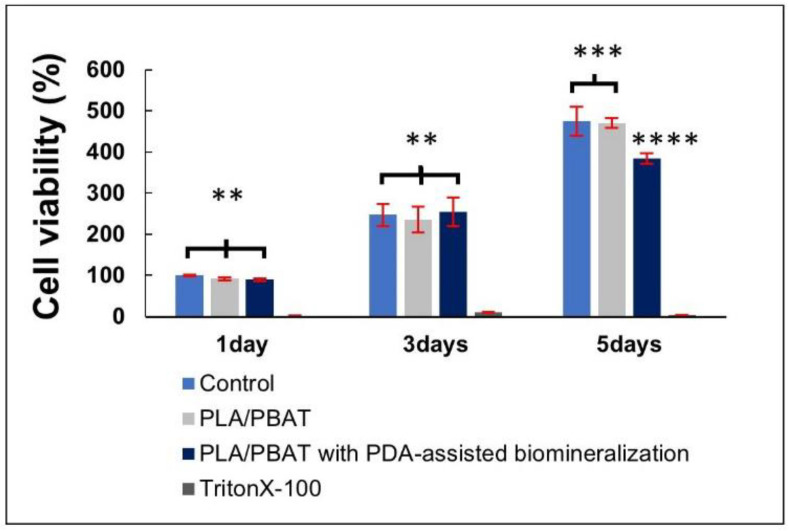
The indirect MTT assay results on the cell viability of MG-63 human osteoblast-like cells. The different numbers of the asterisks represent the statistical significance (*p* ≤ 0.05).

The result of cell viability by MTT assay is presented in Figure 11. The excellent biocompatibility of the PLA/PBAT porous scaffold and the coating material towards the human osteoblast-like cells (MG-63) was confirmed. The pristine PLA/PBAT scaffold did not exhibit a bioactive property toward the human osteoblast cell line. At the fifth day of cell incubation, a significant reduction in the percentage of MG-63 cell viability was observed on cells that were cultured in the extraction medium of PLA/PBAT with PDA-assisted biomineralization.

**Figure 12:**
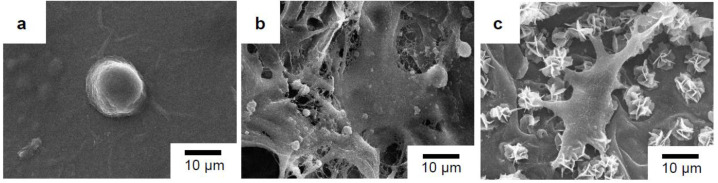
SEM images of MG-63 cell morphology after 3 days of incubation on glass slide as the control (**a**), the pristine PLA/PBAT scaffold (**b**), and the PLA/PBAT scaffold with PDA-assisted biomineralization (**c**).

The cell morphology of the MG-63 cells that were directly seeded on the surfaces of the sterile glass slide and the surfaces of the porous scaffolds with and without PDA-assisted biomineralization after 3 days of incubation is presented in Figure 12. The MG-63 cells, with a round-like shape and some filopodia extensions, were observed when cells were seeded on the surface of the glass slide (Figure 12a) and the pristine PLA/PBAT scaffold (Figure 12b). The alteration of the MG-63 cell morphology to an elongated and flat shape with the spreading of filopodia extension over the surface of the scaffold and biomineralized crystals was seen when cells were seeded on the PLA/PBAT scaffold with PDA-assisted biomineralization (Figure 12c).

#### 3.4.2. The Osteogenesis Property of the Scaffold

**Figure 13:**
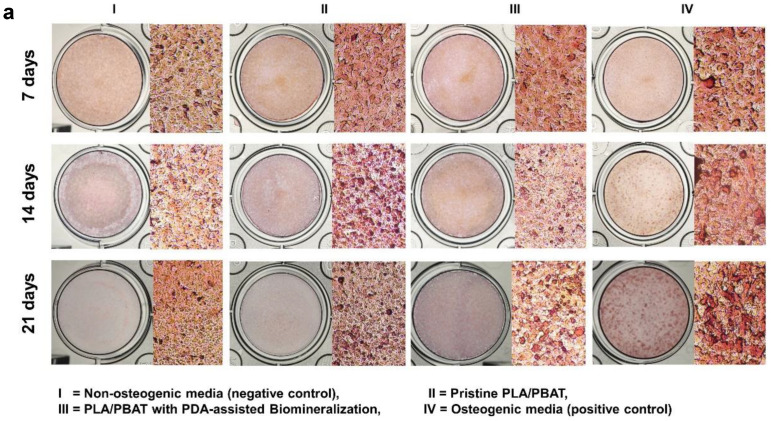

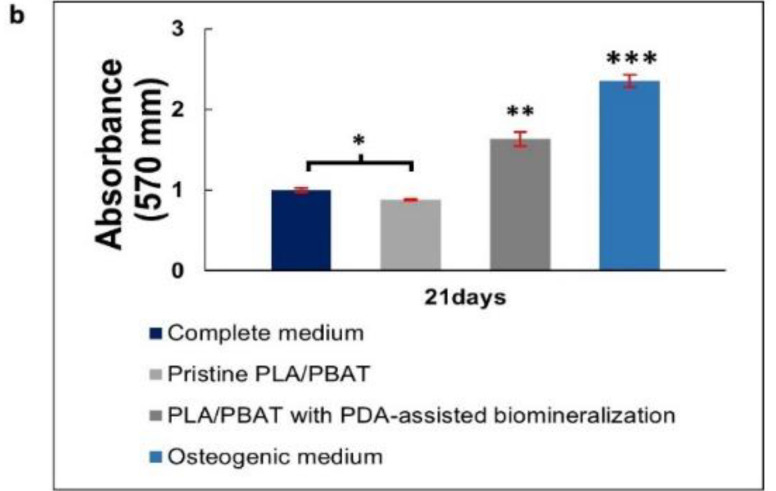
Mineralization assay with Alizarin Red S staining of MG-63 cells. Images of MG-63 cells cultured in the complete medium (negative control), extraction medium of pristine PLA/PBAT, extraction medium of PLA/PBAT with PDA-assisted biomineralization, and the osteogenic media (positive control) for 7, 14, and 21 days (**a**). Quantitative data of Alizarin Red S staining extraction after incubation for 21 days, expressed in the form of absorbance value at 570 nm (**b**). The different numbers of asterisks represent the statistical significance (*p* ≤ 0.05).

Photographs of the Alizarin Red S-stained MG-63 cells cultured in the different extraction media for 7, 14, and 21 days are exhibited in Figure 13a. After incubation for 21 days, it was clear that the darkest staining was observed on the cells cultured with osteogenic medium (positive control), followed by the ones cultured in the extraction medium of the PLA/PBAT scaffold with PDA-assisted biomineralization. 

The amount of cell calcification following incubation for 21 days was investigated and results are presented in Figure 13b. The MG-63 cells cultured in complete media (negative control) showed a non-significant level of calcification compared to the cells cultured in the extraction medium of the pristine PLA/PBAT scaffold. The most intensely calcified tissue was found on the MG-63 cells cultured in osteogenic medium (positive control), followed by the cells cultured in the extraction medium of the PLA/PBAT scaffold with PDA-assisted biomineralization. The statistical significance of cell calcification between the pristine scaffold and the scaffold with PDA-assisted biomineralization was observed (*p* ≤ 0.05). 

## 4. Discussion

In this study, we applied the gas foaming/ammonium bicarbonate particulate leaching technique and PDA-assisted biomineralization surface modification to develop a biodegradable and highly porous PLA/PBAT porous scaffold with osteogenic properties for bone tissue regeneration. The PLA/PBAT binary blend at the ratio of 70:30 wt% was utilized following the recommendation of a previous article [44]. The mentioned authors stated that the addition of 10–50 wt% PBAT to the PLA matrix resulted in an improvement in the impact strength and the elongation at break of the PLA/PBAT blend. However, a sign of brittle-to-ductile transition was notably found when the PBAT ratio was 30 wt%. When the PBAT was incorporated at the ratio of 40 and 50 wt%, the co-continuous phase between the two polymers was observed. The PLA became a dispersed phase in the PBAT matrix. They concluded that the PLA/BPAT blend at the ratio of 70:30 wt% provided the optimum mechanical properties without phase inversion. For these reasons, the PLA/PBAT blend at the ratio of 30:70 wt% was selected to prepare the scaffold in the present study. Two steps of dipping in dopamine solution followed by 10× SBF-like solution were applied to develop the PDA–CaP biomineral composite layer on the surface of the prepared scaffold, to improve the bioactivity and enhance the osteogenic property toward bone cells. The developed scaffold showed strong potential in terms of physicochemical and biological properties for application in bone tissue regeneration.

### 4.1. Scaffold Morphology

Suitable porous architectures and interconnections between each unit, which play essential roles in cell nutrition, tissue vascularization, and new tissue formation, were successfully prepared. The PLA/PBAT scaffold with the total porosity of more than 80% and a well-interconnected porous network throughout the whole scaffold was observed. The differences in the shape, size, and diameter of the opened porous cells inside the scaffold were noted. The combination of macro- and microporous structures was exhibited throughout the prepared scaffold. A macroporous structure with a diameter from 200 to 500 µm, which was within the range of recommend pore sizes for optimal osteoblast proliferation [45], were observed. A previous article explained that a scaffold with a large pore size provides essential advantages in new bone formation by promoting newly formed blood vessels, which are necessary for oxygen and nutrient transportation for osteoblastic activities [46]. In addition, the presence of microporous structures with a diameter ranging from 10 µm to 100 µm in the PLA/PBAT scaffold could provide an advantage in terms of cell seeding efficiency, which affects cell aggregation and proliferation, as mentioned in the previous literature [47]. According to these data, the high porosity, the well-interconnected porous network, and the combination of the macro- and microporous opened porous structures inside the developed PLA/BPAT scaffold could provide an enhancement effect on vascularization and cell behaviors that significantly influences the new bone formation process. The formation of well and evenly distributed porous structures throughout the scaffold could also provide a positive microenvironment that enables living cells to adhere, proliferate, and differentiate in the deep part of the scaffold.

The formation of a dense skin layer on the outer surface of the scaffold, which is usually noticed on specimens prepared by the conventional casting technique, was not found in our scaffold. The combination of the leaching out of the ammonium bicarbonate porogen, as well as the penetration of the carbon dioxide and ammonium gas bubbles through the semi-solidified mixture of polymer/effervescent salt/solvent during the degradation process of the effervescent particles when exposed to heat, played a key role in the even formation as well as the even distribution of the open porous structure and the interconnected networks throughout the whole scaffold [31]. The expansion of the semi-solidified polymer matrix due to the pressure generated by carbon dioxide and ammonium gas could explain the formation of pores with larger diameters than the average size of effervescent particles (200–250 nm). Therefore, the gas foaming/particulate leaching technique that uses ammonium bicarbonate particles as the effervescent porogen could be considered as a simple and facile technique for the fabrication of highly porous specimens with well-interconnected porous networks that are suitable for application in tissue regenerative procedures.

After PDA surface coating, the SEM images at low magnification showed a small difference in the morphological features of the porous structures of the PLA/PBAT scaffold. The small thickness of the PDA-deposited film could be the reason for this finding. The analysis by atomic force microscopy (AFM) in a previous article found that the thickness of the PDA film generated by simple dipping in a dopamine solution at a constant concentration of 2 g/L in the presence of Tris (hydroxymethyl) aminomethane at pH 8.5 was a function of the immersion time and reached a value of up to 50 nm after 24 h [26]. At high magnification, the PDA coating film was made up of a uniform PDA layer and some covalently bonded PDA particles and aggregates. The presence of FTIR peaks at 3388, 1598.94, and 574.68 cm^−1^ that indicated the existence of -OH bending, C-C vibration of the aromatic ring, and N-H bending vibration of the catechol functional groups in PDA molecules confirmed the deposition of the PDA on the scaffold surface.

The significant alteration of the surface morphology was observed following the biomimetic biomineralization. The deposition of the nanoscale plate-like CaP mineralized crystals on the outer surface of the porous structures resulted in the thickening of the porous cell walls and an increase in the surface irregularity, which were clearly observed in the SEM images. This alteration resulted in a significant reduction in the scaffold’s porosity in the PLA/PBAT scaffold with PDA-assisted biomineralization. However, the total porosity of the developed scaffold was still within the range of trabecular bone porosity (75% to 85%) that is suitable for osteogenesis [48]. 

The formation of the CaP biominerals on the surface of the scaffold by the biomimetic biomineralization technique was initiated through the formation of the surface negative charge induced by the hydroxyl (-OH) and amine (-NH_2_) functional groups on the PDA layer. This surface alteration caused the increased concentration of Ca^2+^ ions and the recruitment of hydrogen phosphate (HPO_4_^2−^) ions to the scaffold surface. The Ca^2+^ and HPO_4_^2−^ ions formed an ionic interaction between each other and then resulted in the formation of calcium phosphate nanoparticles. These particles could function as a secondary nucleation site for the newly formed CaP biominerals. According to this mechanism, the continuous growth of the biomineralized layer over time was observed [49]. The efficiency of this mechanism can be accelerated by surface functional groups such as dihydrogen phosphate (-H_2_PO_4_), carboxyl group (-COOH), and methyl groups (-CH_3_), including the hydroxyl group (-OH) and amine group (-NH_2_) that were present on the PDA bioactive layer [50]. In our study, the FTIR spectrum of the PLA/PBAT scaffold with PDA-assisted biomineralization showed peaks that confirmed the existence of PDA and CaP in the biomineralized layer. The XRD pattern also showed peaks that were attributed to hydroxyapatite (210, 211, 203 JCPDS 00-009-0432). These results corresponded to the data from a previous article [22,51]. From these data, the combination of the PDA, crystal phase of HA, and other amorphous CaP components in the biomineralized layer was confirmed. 

The alterations of the porous PLA/PABT scaffold in terms of the surface chemical composition and surface morphology following biomineral deposition could play a significant role in the scaffold–cell interaction. A study on the effect of different biomineralized crystal morphologies and bone cell behaviors [52] revealed a significant enhancement effect on the cellular projection along the coated surface and the higher expression of the mature osteogenic marker in the specimen deposited with a plate-like crystalline structure. Therefore, the formation of the plate-like CaP nanoparticles found on the outer surface of our developed scaffold could also provide an advantage for bone cell responses, besides the osteogenic enhancement due to the presence of the HA-like chemical composition of the CaP biomineralized layer.

The existence of Ca and P elements in the biomineralized layer generated by dipping the PDA-coated scaffold in 10× SBF-solution for 24 h was confirmed. The Ca/P atomic ratio of 2.38, which was higher than the theoretical stoichiometric ratio of 1.67 for hydroxyapatite, was observed in the present study. However, this stoichiometric ratio corresponded to the Ca/P ratio observed on healthy human bone (2.33 ± 0.34) [27]. According to these data, the positive effect on bone cell behaviors of the PLA/PBAT scaffold with PDA-assisted biomineralization could be hypothesized.

Although the similarity of the Ca/P ratio of the biomineralized layer to the stoichiometric ratio of HA was mentioned previously as a significant aspect in preparing the proper calcium-phosphate-based biomaterials for bone regeneration, the other characteristics of the biomineralized layer, such as grain size, porosity, material dissolution rate, and chemical composition, should be considered as other crucial factors that also play a major role in the osteoblast activities [53]. This statement is supported by positive results regarding the cell viability, cell attachment, and osteogenic potential property of the biomineralized layer, which had a different Ca/P atomic ratio compared to the stoichiometric ratio of HA. 

### 4.2. Physicochemical Properties 

#### 4.2.1. Compressive Strength

The deposition of the PDA and CaP biomineralized layer on the PLA/BPAT scaffold surface resulted in a significant reduction in the compressive strength (from 1.072 ± 0.12 MPa to 0.654 ± 0.20 MPa) and the modulus of elasticity (from 0.077 ± 0.017 MPa to 0.05 ± 0.016 MPa). The reduction in the FTIR peaks’ intensity corresponding to PLA and PBAT following the surface modification by PDA and PDA with biomineralization modification in the present study confirmed the formation of the PDA layer over the surface of the porous PLA/PBAT scaffold. The sealing efficiency of the PDA film on the small surface defects and the formation of surface binding at overlapping points of microstructures due to the gluing effect of the PDA film was mentioned in a previous article as the reason for the improvement in the tensile strength and toughness of the porous scaffold with PDA surface coating [43,54]. This explanation could clarify the increasing scaffold elasticity and flexibility following the formation of the PDA film in the present study.

Although the XRD patterns showed peaks that contributed to the HA crystals, the lower intensity of these characteristic peaks indicated the poor formation of the crystals in the biomineralized composite layer. This finding could explain the lower influence of the HA crystals in the biomineralized layer on the compressive strength and modulus of elasticity of the scaffold with biomineralized surface modification found in the present study.

The deformation of the porous scaffold without fracture or breakage under compressive load, as well as the expression of the lower modulus of elasticity following the application of the PDA-assisted biomineralization, are promising for clinical application as the compressible characteristic provides better scaffold adaptability and stability against the recipient sites, especially for defects that are surrounded by bony walls, such as a tooth extraction socket. The primary stability of the scaffold in the defect site during the healing process could favor healing and scaffold integration to the surrounding tissues.

#### 4.2.2. In Vitro Degradation

A significant improvement in the surface contact angle and water uptake was noticed in the PLA/PBAT scaffold with PDA-assisted biomineralization. The presence of the hydroxyl and amine functional groups on the PDA bioactive film facilitated the formation of hydrogen bonding between water molecules, and the material surface could be the reason for the improvement in the surface hydrophilicity of the scaffold with PDA-based surface modification [55]. In the present study, it is important to note that the material’s hydrophilicity was enhanced with PDA alone, and a further improvement was observed when the biomineralized composite layer composed of the PDA and CaP minerals was applied on the scaffold surfaces. This result was consistent with various previous reports [28,30,32], indicating that the addition of the PDA-assisted biomineralization layer enhanced the material’s hydrophilicity. According to the data from FITR spectra, the combination of the PDA molecules and the CaP biominerals in the biomineralized layer generated by the PDA-assisted biomineralization technique was confirmed. For this reason, it was hypothesized that the PDA-assisted biomineralized layer provided hydrophilic amines and hydroxyl groups due to the exposure of the PDA molecules at the biomineralized surface. Moreover, the surface morphology alteration following the deposition of the biomineralized layer could also influence the increase in the material’s hydrophilicity, as it is generally accepted that the increasing surface roughness on the hydrophilic surface provides a significant improvement in the surface hydrophilicity [56]. Therefore, the presence of functional groups on PDA molecules, and the increasing surface irregularity and roughness following surface mineralization, could be the key factors that provide a synergistic effect toward the improvement of the material’s hydrophilicity in the PLA/PBAT scaffold with PDA-assisted biomineralization.

The agreement between the water absorption value of the pristine scaffold and the scaffold with PDA-assisted biomineralization observed in the present study confirmed the effect of the other factors that played a more significant role in the water absorption of the porous scaffold, besides the formation of the PDA-assisted biomineralization layer. It could be hypothesized that the high percentage of porosity and interconnected porous structures in the material matrix enhanced the water entrapment through capillary action and played a major role in the water absorption capacity of the scaffold [57]. According to these data, the hydrophilic property of the porous PLA/PBAT scaffold with PDA-assisted biomineralization was confirmed. This property was reported as one of the crucial factors in terms of cell adhesion and other cellular behaviors that are related to bone regeneration [58].

The small decrease in the soaking medium’s pH from 7.33 to ~7.2 following the in vitro degradation test for 30 days indicated the progression of the hydrolysis reaction of the PLBA/PBAT blend [59] in the present study. The acidic products generated from PLA hydrolysis, mainly lactic acid, explained the decrease in the SBF solution pH. However, the pH alteration observed following the in vitro degradation test for 30 days was still within the range of pH that is suitable for the proliferation of mammalian cells (7.0 to 7.2) [60]. 

#### 4.2.3. Chemical Composition 

The functional groups of the PLA/PBAT with PDA-assisted biomineralization were characterized by ATR–FTIR. The broad absorbance at 3380.60 cm^−1^ and the peaks at 1600 cm^−1^, which were assigned to -NH/-OH stretching vibrations, and the amide -NH shearing vibration of the polydopamine [52,53], including the characteristic peaks at 600 and 560 cm^−1^, which belonged to the PO_4_^3−^ from hydroxyapatite [61], were observed. This finding confirmed the formation of the organic–inorganic bioactive layer that comprised polydopamine and the CaP biomineral on the surface of the PLA/PBAT scaffold with PDA-assisted biomineralization. The obvious reduction in the FTIR peaks’ intensity at 1754.90 cm^−1^ that contributed to the stretching vibrations of the C=O group of PLA following the deposition of the PDA film and PDA-assisted biomineralized layer indicated the interaction between the carbonyl group (C=O) on the polymer surface and the catechol functional group of PDA molecules [62]. This finding confirmed the deposition of the PDA layer on the surface of the PLA/PBAT scaffold.

The XRD pattern showed sharp peaks at 31.8° and 45.6° following the PDA-assisted biomineralization process that were related to the HA characteristic peaks (211) [63] and (222) [22], respectively. These peaks confirmed the existence of the crystalline component in the composite biomineralized layer.

According to these data, we can state that the biomimetic biomineralized layer generated by two steps of simple dipping in dopamine solution followed by 10× SBF-like solution for 24 h was composed of PDA molecules, HA crystals, and other CaP mineral components. The deposition of the biomineralized particles on the PLA/PBAT surface was evidenced by SEM, EDX, FTIR, and XRD. The reduction in the FTIR spectra’s peak intensity corresponded to the carbonyl groups of PLA molecules that contributed to the bonding between the catechol functional groups of the PDA molecules in the PDA/HA/CaP biomineralized layer and the polymer matrix surface.

### 4.3. Biological Properties

The excellent biocompatibility of the pristine PLA/PBAT porous scaffold and the biomineral coating material toward the human osteoblast-like cells (MG-63) was observed. The absence of the biological enhancement effect of the pristine PLA/PBAT scaffold toward the human osteoblast cells was confirmed, as reported in a previous study [17]. The remarkable inverse relationship between cell proliferation and cell differentiation could explain the significant reduction in MG-63 cell viability following the 5 days of cell culture in the extracted medium of PLA/PBAT with PDA-assisted biomineralization, compared to the group that was incubated in the extracted medium of pristine PLA/PBAT. According to the literature, the cell differentiation process starts with the continuous division of the precursor cells at the initial stage, while the terminal differentiation process usually coincides with the arrest of proliferation and permanent exit from the division cycle [64]. Therefore, the application of PDA-assisted biomineralization on the surface of the porous PLA/PBAT scaffold could result in an improvement in the osteogenicity of the inert PLA/PBAT scaffold.

Generally, the osteogenic differentiation process is classified into three main stages, namely cell proliferation, extracellular matrix (ECM) formation, and matrix mineralization [65]. The adhesion of cells on the scaffold surface is the first critical step for the progression of cell proliferation [66]. The SEM images of the present study clearly exhibited the efficient attachment of human osteoblast-like cells (MG-63) on the surface of the porous PLA/PBAT scaffold coated with the biomineralized layer. The adhered cells on the surface of the PLA/PBAT scaffold with PDA-assisted biomineralization showed a significant alteration in cell morphology compared to the control. This finding indicated the enhancement effect of the biomineralized layer on osteoblast cells’ proliferation, which was related to the first stage of the osteogenic differentiation process.

The formation of the extracellular matrix’s mineralization is considered as a marker of the final stage of osteogenic differentiation. In the present study, Alizarin Red staining showed a significantly higher level of mineralization when the MG-63 cells were cultured in the extraction medium of the PLA/PBAT scaffold with PDA-assisted biomineralization. This finding clarified the positive effect of the biomineralized layer on the late stage of the osteogenic differentiation process.

The positive effects on the cell adhesion and ECM mineralization of the porous PLA/PBAT scaffold with PDA-assisted biomineralization could be explained by the presence of Ca and P components in the deposited biomineralized layer. The formation of the Ca^2+^ and PO_4_^3−^ ions by the dissolution of the CaP biomineralized layer provided essential effects on bone cell metabolic activities and behaviors. The presence of Ca^2+^ provided enhancement effects on cell adhesion, cell proliferation, and cell differentiation. In addition, the released PO_4_^3−^ ions provided an essential effect by regulating cell proliferation and stimulating the proteins related to ECM mineralization [67]. According to these data, the positive effect of the composite PDA-assisted biomineralized layer on the osteogenic improvement of the developed porous PLA/PBAT scaffold was confirmed. 

## 5. Conclusions

In summary, the biocompatible and highly porous PLA/PBAT scaffold with well-interconnected networks was successfully prepared by a simple and effective technique, gas foaming/ammonium bicarbonate particulate leaching. The surface modification by two steps of simple dipping in dopamine and 10× SBF-like solution succeeded in depositing the composite organic–inorganic biomineralized layer on the porous architecture of the PLA/PBAT scaffold. Improvements in surface hydrophilicity, material bioactivity, and osteogenic effects toward human osteoblast-like cells (MG-63) were observed following the application of PDA-assisted biomineralization. The formation of plate-like apatite crystals and CaP mineral nodules on the scaffold surface created a microenvironment that favored the osteoblast cells’ adherence, proliferation, and differentiation. According to the data in the present study, the porous PLA/PBAT scaffold with PDA-assisted biomineralization exhibits strong potential for use as a bone scaffold or alternative bone biomaterial for tissue engineering.

## Figures and Tables

**Table 1:** Components of 10× SBF-like stock solution for a total volume of 1000 mL [32].

Reagent	Order	Amount (g)	Concentration (mM)
NaCl	1	58.443	1000
KCl	2	0.373	5
CaCl_2_·2H_2_O	3	3.675	25
MgCl_2_·6H_2_O	4	1.016	5
NaH_2_PO_4_	5	0.25	3.62
NaHCO_3_	6	0.84	10

## Data Availability

The data used to support the findings of this study are available from the corresponding author upon request.
